# Coupling Demographic and Genetic Variability from Archived Collections of European Anchovy (*Engraulis encrasicolus*)

**DOI:** 10.1371/journal.pone.0151507

**Published:** 2016-03-16

**Authors:** Paolo Ruggeri, Andrea Splendiani, Cristina Di Muri, Tatiana Fioravanti, Alberto Santojanni, Iole Leonori, Andrea De Felice, Ilaria Biagiotti, Piera Carpi, Enrico Arneri, Paola Nisi Cerioni, Massimo Giovannotti, Vincenzo Caputo Barucchi

**Affiliations:** 1 Dipartimento di Scienze della Vita e dell’Ambiente, Università Politecnica delle Marche, Via Brecce Bianche, 60131 Ancona, Italy; 2 Consiglio Nazionale delle Ricerche, Istituto di Scienze Marine Sezione Pesca Marittima, Largo Fiera della Pesca, 60125 Ancona, Italy; 3 FAO-FIRF, Fisheries and Aquaculture Department, AdriaMed Project, Viale delle Terme di Caracalla, 00153 Roma, Italy; College of Charleston, UNITED STATES

## Abstract

It is well known that temporal fluctuations in small populations deeply influence evolutionary potential. Less well known is whether fluctuations can influence the evolutionary potentials of species with large census sizes. Here, we estimated genetic population parameters from as survey of polymorphic microsatellite DNA loci in archived otoliths from Adriatic European anchovy (*Engraulis encrasicolus*), a fish with large census sizes that supports numerous local fisheries. Stocks have fluctuated greatly over the past few decades, and the Adriatic fishery collapsed in 1987. Our results show a significant reduction of mean genetic parameters as a consequence of the population collapse. In addition, estimates of effective population size (*N*_e_) are much smaller than those expected in a fishes with large population census sizes (*N*_c_). Estimates of *N*_e_ indicate low effective population sizes, even before the population collapse. The ratio *N*_e_/*N*_e_ ranged between 10^−6^ and 10^−8^, indicating a large discrepancy between the anchovy gene pool and population census size. Therefore, anchovy populations may be more vulnerable to fishery effort and environmental change than previously thought.

## Introduction

Population dynamics are driven by a complex set of ecological and evolutionary variables that mold population demography on several spatial and temporal scales [1]. Unfortunately, a temporal dimension is not always considered in the assessment of population structure, because historical samples are not always available, or because of the high costs of retrospective laboratory analysis [reviewed in 2]. Among vertebrates, populations of bony fishes are most often examined for historical trends because of the availability of historical tissues [3], which are sometimes available from archeological excavations [3] and from archived fish scales and otoliths used for aging by fishery managers [4]. The extraction of DNA from these archived collections (archived DNA) offers the opportunity to investigate the temporal dynamics of fish populations [2].

Several key variables influencing the evolutionary potential of fish populations and conservation status can be evaluated using archived DNA [5]. This approach has successfully detected the loss of genetic variability as a consequence of natural fluctuations in population census size (*N*_c_) [2, 3, 5]. Many marine fishes with large *N*_c_ are thought to be sheltered from rapid genetic collapse because of large population sizes, but recent genetic studies cast doubt on this conclusion [6, 7]. Temporal genetic analyses of archived samples have also demonstrated that fishing can lead to the loss of allelic richness and heterozygosity in over-exploited marine populations, indicating that overharvesting can drive the loss of evolutionary potential [8–11]. The analysis of archived DNA can provide estimates of effective population size (*N*_e_), “the size of an ideal population (i.e., with discrete generations, random mating, and constant population sizes) that would undergo the same amount of genetic drift, measured by the rate of loss of heterozygosity, as the actual population” [12]. *N*_e_ is a key conservation parameter, which represents the “genetic currency” of evolutionary potential of a population [2, 13]. *N*_e_ measures the extent of genetic erosion by genetic drift and, together with life history traits of a species, can predict future population viability [13].

Among marine species, small pelagic fish periodically experience rapid fluctuations in *N*_c_ [14, 15], but the mechanisms leading to the rapid loss of individuals are still uncertain [16, 17]. Many stocks of small pelagic fishes are the targets of intensive fishing throughout the world. Unfortunately, how fishing activities influence local fluctuations in *N*_c_ and the effect on the evolutionary dynamics of local populations are not fully understood [8]. The Mediterranean area is one of the most exploited in the world with over 80% of its stocks are facing overexploited [18]. In recent years, European anchovy (*Engraulis encrasicolus*, hereafter “anchovy”) stocks were over-exploited in two Spanish GSAs (Geographical Sub-Areas) (GSA 1 and GSA 6) [18], and signs of overfishing were found in the northern Adriatic Sea (GSA 17) [19]. Adriatic anchovy populations have experienced strong fluctuations over the past four decades [20–23]. A decline, beginning in 1978, led to a stock collapse in 1987, with a recent partial recovery [24]. The 1987 stock collapse was preceded by low recruitment in 1986 and 1987 [25]. Recruitment failure has been suggested for the anchovy stock collapse, but the effects of intense fishing pressure cannot be excluded [21, 26]

Recent genetic studies of small pelagic fishes other than anchovies show that temporal variation in *N*_e_ and genetic diversity is often lower than previously believed [6, 27]. These studies suggest that both fishing and other factors, such as environment shifts and interactions with other species, can lead to the loss of genetic diversity [8]. The evaluation of genetic variables can help to understand the status and factors influencing the short-term standing variation of European anchovies in the Mediterranean Sea. The goal of this study is to assess temporal genetic variation in European anchovies with a survey of microsatellite DNA extracted from archived otoliths collected over the past four decades at two sites in the Adriatic Sea. The results were used to address two questions: i) whether the large fluctuations in *N*_c_ experienced by Adriatic anchovies produced a critical loss of genetic diversity that could lead to a loss in evolutionary potential; and ii) whether genetic population structure changed in response to the demographic fluctuations.

## Materials and Methods

### Samples studied

**Fig 1:**
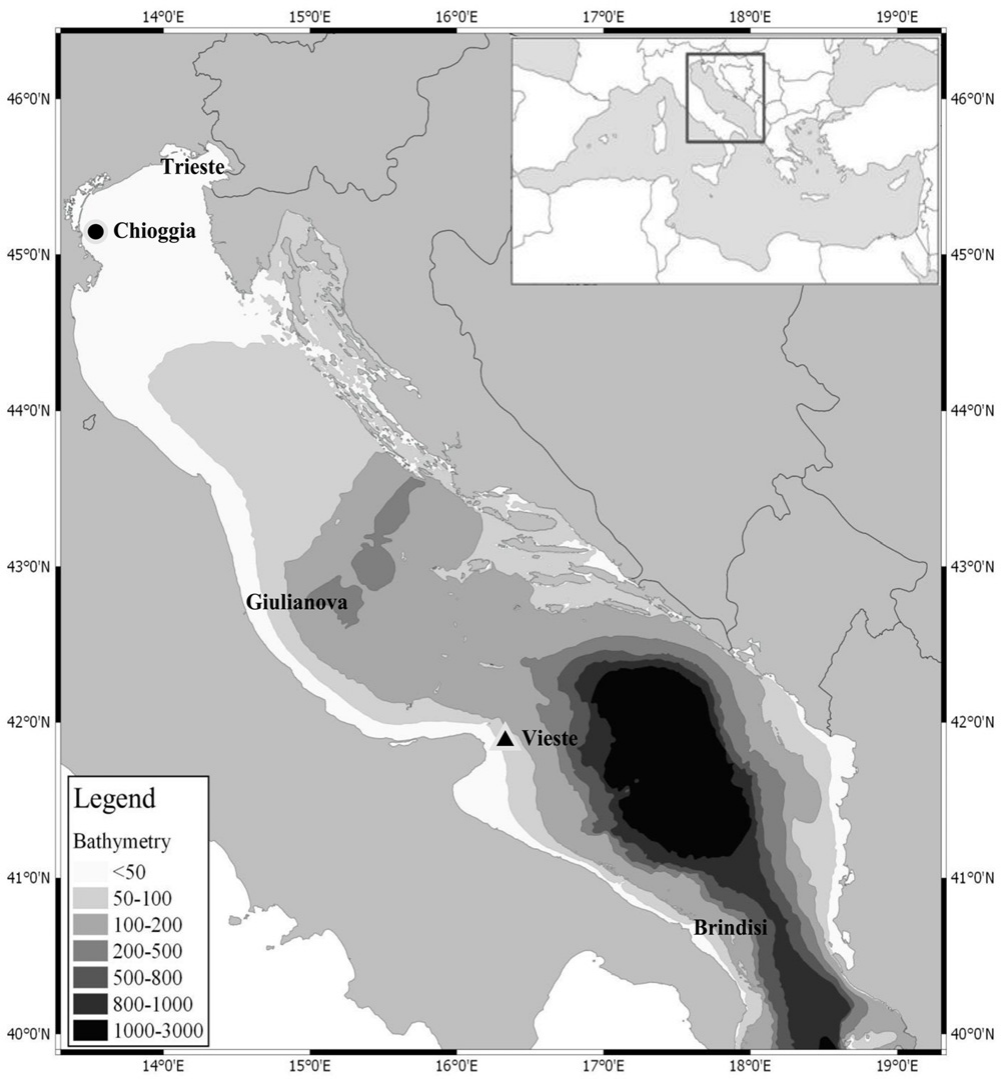
Sampling area. The solid circle indicates the location of the Chioggia time series between 1978 and 2010. The solid triangle indicates the locations of the Vieste time series between 1985 and 2010.

We used 408 archived anchovy specimens, each providing two otoliths. Samples were provided by the ISMAR–CNR of Ancona, Italy (Istituto di Scienze Marine–Consiglio Nazionale delle Ricerche) and were collected 1978–2000. After removal from individuals for age determination, otoliths were stored individually in plastic tubes at room temperature. We analyzed a time series of samples from two spawning grounds, the northern Adriatic (Chioggia) from 1978, 1987, 1994 and 2000, and the middle-southern Adriatic (Vieste) from 1985, 1987 and 1989 (Fig 1). Samples from these years were chosen to include the biomass fluctuations starting in the mid 1970s. This objective was easily fulfilled for samples from Chioggia, because of a more complete time series. Unfortunately, the Vieste time series was available for only 1985–1989. We used otoliths collected between May and September to include individuals collected only during the spawning period. These time series were compared with 73 contemporary fish collected in 2010 in surveys off Chioggia and Vieste. The number of specimens used for each sampling year and location appears in S1 Table.

### Ethics statement

Ethical procedures were not required to manipulate specimens in this study. Tissues used in this study were recovered from previously collected otoliths and from Italian commercial catches.

### Genomic DNA extraction

Genomic DNA was extracted from tissue residuals on otolith following the method described in [28]. Each set of otoliths was incubated at 55°C (up to 5 hours) in 500 μL of digestion buffer (100 mmol·L^–1^ Tris–HCl, pH 8.0; 100 mmol·L^–1^ NaCl; 1 mmol·L^–1^ EDTA, pH 8.0; 0.5% SDS). EDTA and SDS concentrations in the digestion buffer were reduced according to [29]. Fin or caudal muscle tissues from contemporary samples were used for DNA extraction by standard phenol–chloroform procedures described in [30].

### PCR amplification and genotyping

Samples were screened at 7 microsatellite loci that yielded short alleles less than 200 bp, a feature enhancing the ability to amplify degraded DNA from archived tissues. Primers for 6 of these loci were obtained from the European anchovy genome described by [31] (Ee10) and [32] (Ee2-508, Ee2-165b, Ee2-135, Ee2-407 and Ee2-91b). Primers for the seventh locus (Eja183) was described by [33] in the Japanese anchovy (*Engraulis japonicus*) genome (S2 Table). However, new primers were needed to obtain molecular sizes smaller than those produced by the original primers. Hence, primer sequences for these loci, except Ee2-91b and Ee2-135, were re-designed using the online program Primer3Plus (http://www.bioinformatics.nl/cgi-bin/primer3plus/primer3plus.cgi/) [34] from their NCBI (National Center for Biotechnology Information) clone sequence references (S2 Table). Attempts to develop additional primers producing shorter alleles to increase the number of loci failed because sequence constraints did not allow the design of new primers.

PCR conditions were optimized for all loci using touchdown amplification. The PCR reaction mixture contained approximately 5–10 ng genomic DNA, 0.25 U Taq DNA polymerase (MyTaq, Bioline), 0.5 μmol·L^–1^ of each primer, and 1× MyTaq (Bioline) Reaction buffer (15 mmol·L^–1^ MgCl_2_, 1.25 mmol·L^–1^ of each dNTP, plus stabilizers and enhancers) in a total volume of 10 μL. Each PCR plate included a blank control, including an extra control from each DNA extraction. PCR products were separated on a 2% (w/v) agarose gel and stained with GelRed^™^ (Biotium, Inc.) to check for size and PCR specificity. Subsequently, these products were run on a 5% denaturing polyacrylamide sequencing gel and visualized by a silver staining protocol [35]. Genotyping procedures were described in [6].

### Accuracy of extraction and genotyping archived DNA

Archived DNA is particularly prone to genotyping errors due to i) its degraded nature increases the risks for allele dropout and null alleles and ii) potential contamination from exogenous DNA. To address these issues, a standardized protocol was used for DNA extraction and genotyping. First, archived DNAs were extracted in an ancient-DNA room equipped with sterilized laboratory utensils regularly sterilized and supplies dedicated to analyzing archived materials. Secondly, DNA extractions were performed in clusters of individuals from the same locality and year. These precautions helped to avoid potential contamination of DNA between individuals from different years and sampling sites. To further reduce sample contamination, no contemporary anchovy samples were processed while working on archived samples.

Genotyping errors occur primarily during PCR amplification by increasing the number of DNA copies from non-target individuals. Hence, PCRs were carried out in a separate ancient-DNA room, furnished with PCR workstations, set of pipettes, reagents and thermal cyclers used exclusively to amplify archived DNA. PCRs were carried out in single tubes and individuals were amplified in clusters belonging to the same sampling site and year. Additional attention was paid to the number of individuals amplified per PCR; less than 1/4 of the capacity of each thermal cycler was used to maximize the space between tubes. Thermal cyclers were sterilized by UV light and by cleaning with 10–20% bleach before PCR.

Contamination was tested by screening PCR products from two microsatellite loci, Ee2-91b and Ee2-165b. These loci show clear allelic patterns and were used to eliminate individuals that showing multiple alleles indicating contamination (S1 Table). Faint genotypes from uncontaminated individuals were initially entered as missing data. DNA from these individuals was amplified three times using a progressively stringent PCR protocol. This was accomplished by increasing the concentration of Taq Polymerase in each reaction (0.05 μl, 0.1 μl, 0.2 μl on a final volume of 10 μl) and by increasing the annealing temperature by 0.5°C per PCR. Genotypes were not clearly detectable after these three trials were excluded from the dataset. A set of 10 individuals were randomly selected from each site and genotyped again as quality controls.

Finally, the probability of identity, *P*_ID_, and probability of siblings, *P*_ID(sib)_, were used to test whether contamination biased genotype quality of our dataset. *P*_ID_, the probability that two unrelated individuals in a dataset will have the same multilocus genotype by chance, and *P*_ID(sib)_, an analogous that takes inbreeding into account, were calculated with GenAlEx 6.502 [36]. Since we analyzed years and sampling locations independently, we assumed that DNA contamination would only be by individuals in the same sample. Therefore, cross-contaminated DNA should share more common genotypes than non-contaminated DNA. Thresholds for detecting contaminated samples were set at *P*_ID_ *>* 0.001–0.0001 and *P*_ID(sib)_ > 0.01, as proposed by [37].

### Statistical treatment of data

We estimated genetic diversities from genotypic and allelic frequencies only after checking the quality of the genotypes (amplification success rate and genotype consistency) and the occurrence of null alleles and other genotyping errors (allele dropout and stutter peaks) using MICRO-CHECKER 2.2.1 [38]. Loci affected by null alleles were corrected following the Brookfield algorithm [39]. Mean number of alleles observed for each locus (*N*_A_), allelic richness (*R*_S_), observed (*H*_o_) and expected (*H*_e_) heterozygosities, and the inbreeding coefficient (*F*_IS_) were estimated with FSTAT 2.9.3 [40]. Linkage disequilibrium between loci was tested with a Monte Carlo Markov Chain (MCMC) test executed by 1000 batches of 2000 iterations each, using Genepop 4.0.10 [41]. A sequential Bonferroni adjustment of P-values was applied to account for an increase in type-I error from multiple comparisons [42]. Outlier loci were detected by departures from the neutral expectations with *fdist* [43] implemented in Lositan [44] and a LnRH method developed for microsatellites [45] (S1 File).

Temporal variation in genetic diversity was quantified using estimates of expected (*H*_e_) and observed (*H*_o_) heterozygosity and the expected number of alleles (*N*_A_). Since estimates of genetic diversity can be biased by differences in sample sizes, the software POPTOOLS 3.1.0 [46] (http://www.cse.csiro.au/poptools) was used to standardize and analyze temporal trends in these variables. Estimates were standardized by generating 1000 'real' samples (n = 24) with sampling without replacement for each year and location (Chioggia and Vieste). A total of 9000 “real” samples were generated. In addition, 9000 “randomized” samples were generated pooling together the samples from the Chioggia and Vieste time series. Statistical significances of temporal trends were estimated by calculating the slope (b) and the Pearson’s correlation coefficient (r) of linear regressions of *H*_e_, *H*_o_, and *N*_A_ against years. The r and b estimates of "real" parameters for each year in both the Chioggia and Vieste time series were compared with randomized values, using MCMC chains of 1000 iterations. We evaluated the significance of trends in *H*_e_, *H*_o_, and *N*_A_ over the entire time-series and between consecutive years.

Effective population size (*N*_e_) was estimated using a moment-based temporal method and a coalescent likelihood-based method. These methods are generally robust and are often used on historical time series [13]. Both temporal methods use changes in allele frequencies between two samples separated by a known number of generations. Since anchovies become mature at one year of age [24], one generation per year was assumed. Following [47], *N*_e_ was also estimated over the whole timeframe of each time series to detect consistency with estimates obtained from intermediate temporal timeframes. NE-ESTIMATOR 2.01 [48] was used to estimate moment-based *N*_e_ values. We implemented the following procedures: i) we used Plan II sampling in which individuals were removed after sampling, ii) we used the Pollak formula [49] for computing the standardized variance in allele frequency (*F*_K_) to estimate *N*_e_ values, iii) we estimated 95% confidence intervals from jackknifing, and iv) we used a threshold of 0.01 as the lowest allele frequency considered to estimate *N*_e_ values, because allele frequencies were seldom lower than 0.01. The TM3 software [50] was used to obtain coalescent likelihood-based estimates of *N*_e_. TM3 uses a Bayesian approach based on coalescent theory and MCMC simulations to generate a posterior distribution of *N*_e_ values. In addition, we used maximum *N*_e_ values (Ne MAX) and the number of generations between consecutive samples (T) as priors in the TM3 simulations. To test for consistency between simulations, three independent runs were performed using Ne MAXes equal to 1000, 5000, and 10,000. Simulations were performed with 20,000 MCMC iterations and were conducted separately for Chioggia and Vieste samples.

**Fig 2:**
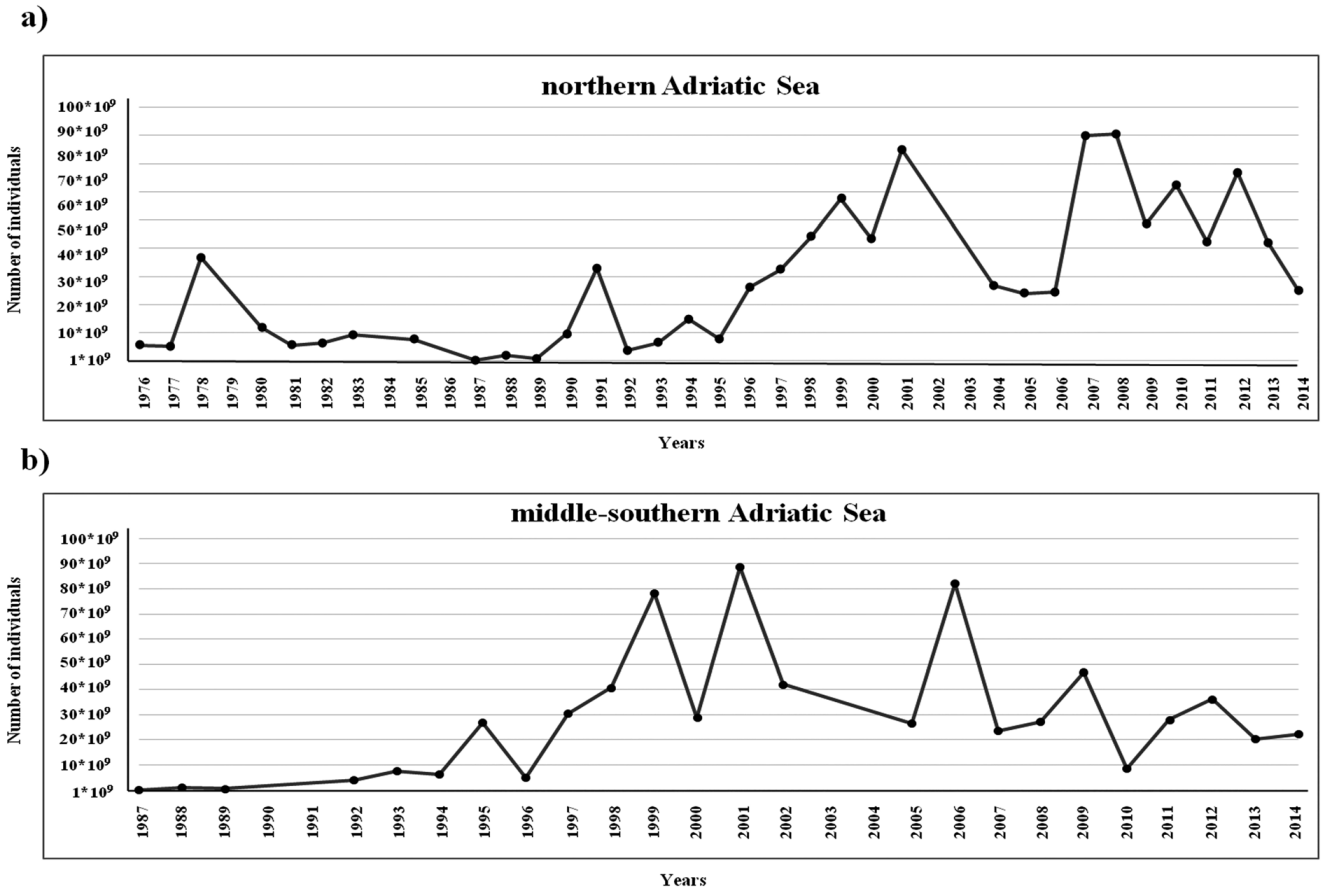
Graphical representation of temporal trends of anchovy population abundance (expressed as number of individuals) obtained from annual MEDIAS acoustic surveys data (black dots). (**a**) trend of abundance in the northern Adriatic Sea. (**b**) trend of abundance in the middle-southern Adriatic Sea.

Estimates of *N*_e_ from the moment-based temporal method were used to provide temporal trends in *N*_e_/*N*_c_ ratios from both time series. Estimates of *N*_c_ were obtained from annual acoustic surveys (MEDIAS Project, http://www.medias-project.eu; Fig 2) [22, 23] for stocks in the northern and middle-southern Adriatic Sea.

Datasets were tested for genetic population bottleneck signatures with a heterozygosity-excess based method implemented in BOTTLENECK 1.2 [51]. This test assumes that during a strong reduction in population size, allele numbers decline faster than *H*_e_. Since expected heterozygosity at mutation–drift equilibrium (*H*_eq_) is calculated from the number of observed alleles, *H*_e_ becomes larger than *H*_eq,_ leading to heterozygosity excess (*H*_exc_) [52]. Values of *H*_exc_ were tested with Wilcoxon’s signed rank tests [53], a powerful and robust test when the dataset contains few (< 20) polymorphic loci [51]. We used 1000 iterations and three mutational models: infinite allele model (IAM), stepwise mutation model (SMM), and a two phase mutation model (TPM) with 95% single-step mutations and 5% multistep mutations, as recommended in [51].

In addition, purported bottlenecks were verified by simulating bottlenecked populations using BOTTLESIM 2.6 [54]. Genotypic data for CH78 and VI85 were used independently as founder populations to simulate bottleneck events lasting 32 and 25 generations, respectively. Population declines were tested using 1000 iterations with the following criteria: dioecious organisms with random mating system, balanced sex ratio (1:1), two years of expected lifespan for the species, given its high natural mortality [19], one year at first reproduction [24], and 90% generational overlap. The moment-based *N*_e_ estimates from NE-ESTIMATOR were used as analogues of population size. Simulated values of *H*_e_ and *N*_A_ were plotted and compared with observed *H*_e_ and *N*_A_ estimates to provide evidence of a population bottleneck.

Finally, we estimated the degree of geographic and temporal variability among samples first with pairwise values of θ_ST_ [55], calculated with FSTAT and second with a Bayesian approach, implemented in STRUCTURE 2.3.2.1 [56, 57]. The second approach estimates the number of populations (*K*) under Hardy–Weinberg expectations and linkage equilibrium. The most probable number was tested using priors ranging from *K* = 1 to *K* = 6, under an admixture model and with correlated allele frequencies. Ten independent runs were performed for each *K* using an MCMC of 500,000 iterations after a burn-in of 50,000 iterations.

## Results

Missing genotypes accounted for 7.51% of the overall dataset. A pool of 90 individuals were randomly chosen (10 individuals per sampling year and site) to re-genotype 7 of the screened loci (a total of 630 control genotypes were produced). We were unable to PCR-amplify 2.3% of control genotypes (15 of 630), but the remaining 615 genotypes were consistent with their first molecular size detection. There was a lack of allele dropout and stuttering throughout the entire dataset. The occurrence of null alleles was detected in 16 of 63 tests, and after the Brookfield correction [39] 6 of 63 remained significant. Since the Brookfield correction improved our dataset, and no loci showed a systematic presence of null alleles, the entire corrected dataset was used for subsequent statistical analyses.

*P*_ID_ values ranged from 7.731 x 10^−9^ (CH87) to 2.854 x 10^−7^ (CH94), and *P*_ID(sib)_ values ranged from 1.133 x 10^−3^ (CH87) to 2.743 x 10^−3^ (CH94) (S3 Table). Both values were lower than expected thresholds for unrelated or unbiased multilocus genotypes, suggesting enough power and sufficient quality of the data.

The observed number of alleles ranged from 7 (Ee2-165m) to 38 (Ee2-407m) across samples. Mean *N*_A_ ranged from 9.143 (CH10) and 12.000 (VI89 and VI10), whereas *R*_S_ ranged from 6.982 (CH94) to 8.769 (VI87). Mean *H*_e_ ranged from 0.703 (CH94) to 0.767 (CH87 and CH10), while mean *H*_o_ varied from 0.590 (CH87) to 0.746 (CH00) (S4 Table). There were 4 of 63 significant *F*_IS_ values (critical P < 0.00079). Two deviations were related to the Ee2-165m locus and led to heterozygote excesses in samples CH94 and CH00, while the remaining two were related to the Ee2-407m locus and suggested heterozygote deficiency in CH87, and VI87 (S4 Table). Significant heterozygote deficits were found in samples CH78, CH87, and VI10 (S4 Table), and for the Ee2-407m locus overall. No evidence for linkage disequilibrium was detected.

**Fig 3:**
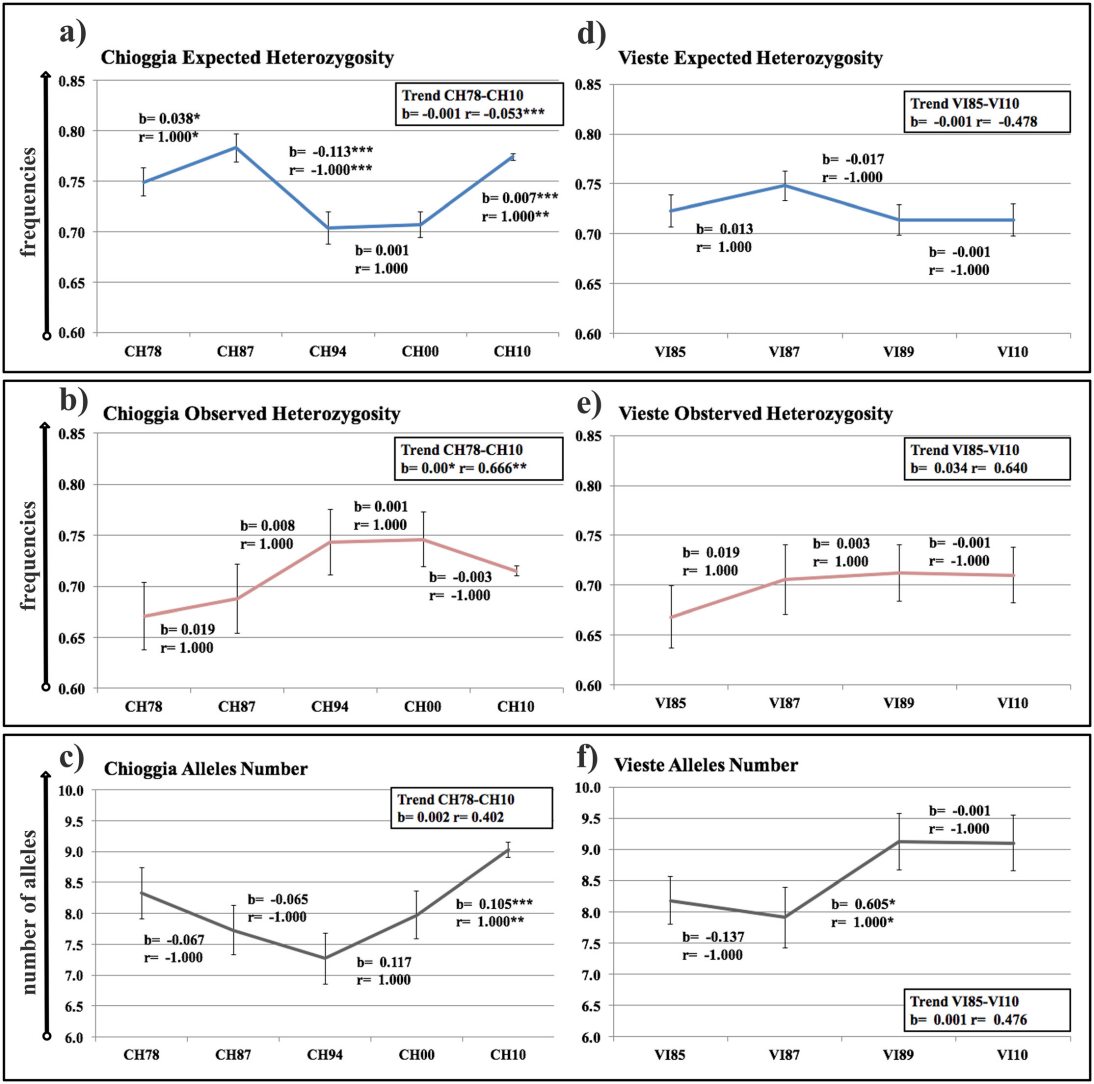
Temporal changes in estimates of expected (*H*_e_) and observed (*H*_o)_ heterozygosities, and mean number of alleles (*N*_A_). Panels (**a**) (*H*_e_), (**b**) (*H*_O_) and (**c**) (*N*_A_) depict temporal trends at Chioggia, and panels (**d**) (*H*_e_), (**e**) (*H*_o_) and (**f**) (*N*_A_) depict temporal trends at Vieste. Standard deviation intervals are provided for each sample, estimated from 1000 resampled samples per year and standardized at 24 individuals. Statistical significances of *b* (slope) and *r* (Pearson’s regression coefficient) indicate global trends (into the box to the left of each panel) and trends from consecutive temporal samples (above or below each timeframe considered). Level of significance: * *P* < 0.05; ** *P* < 0.01; *** *P* < 0.001.

The overall temporal trends in *H*_e_, *H*_o_, and *N*_A_ of the Chioggia time series indicated a significant overall decrease in *H*_e_ (*r* = -0.053, *P* < 0.001; *b* = -0.001, *P* > 0.05), a significant overall increase in *H*_o_ (*r* = 0.666, *P* < 0.01; *b* = 0.002, *P* < 0.05), and a non-significant overall increase in *N*_A_ (*r* = 0.402, *P* > 0.05; *b* = 0.022, *P* > 0.05) (Fig 3a, 3b and 3c). Specifically, a highly significant decreasing trend was observed in *H*_e_ between CH87 and CH94 (Fig 3a; *r* = -1.000, *P* < 0.001; *b* = -0.113, *P* < 0.001), whereas significant increasing trends were observed in *H*_e_ between CH78–CH87 (Fig 3a; *r* = 1.000, *P* < 0.05; b = 0.038, *P* < 0.05) and between CH00-CH10 (Fig 3a; *r* = 1.000, P < 0.01; b = 0.007, *P* < 0.001), as well as, in *N*_A_ between CH00–CH10 (Fig 3c; *r* = 1.000, *P* < 0.01; *b* = 0.105, *P* < 0.001). The Vieste time series showed a non-significant overall decrease in *H*_e_ (*r* = -0.474, *P* > 0.05; *b* = -0.001, *P* > 0.05), a non-significant overall increase in *H*_o_ (*r* = 0.481, *P* > 0.05; *b* = 0.001, *P* > 0.05) and a non-significant overall increase in *N*_A_ (*r* = 0.640, *P* > 0.05; *b* = 0.034, *P* > 0.05) (Fig 3d, 3e and 3f). Uniquely, the VI87–VI89 trend showed a significant increase in *N*_A_ (Fig 3f; *r* = 1.000, P < 0.05; *b* = 0.605, *P* < 0.05).

Moment-based estimators yielded *N*_e_ values between 103.50 and 416.70 from the Chioggia time series (Table 1) and values between 68.00 and 891.20 from the Vieste time series (Table 1). Estimates from the overall timeframes showed *N*_e_ values of 597.70 in Chioggia and 666.70 in Vieste (Table 1).

**Table 1:** Temporal moment-based effective genetic size estimates. *N*_G_ = number of generations between a pair of samples. *N*_e_ = effective genetic size estimate. Lower and upper 95% CI represents a 95% Confidence Interval (CI) for *N*_e_ estimated for each pair of samples. *N*_e/_*N*_c_ ratio = ratio between effective population size (estimated from temporal moment-based method) and census size (obtained from MEDIAS acoustic survey data; Fig 2). NA = not available data.

Timeframe	N_G_	*N*_e_	Lower 95% CI	Upper 95% CI	*N*_e/_ *N*_c_ ratio	
CH78-87	9	167.00	104.00	284.50	1.90*10^−8^ CI (1.18*10^−8^–3.23*10^−8^)	
CH87-94	7	103.50	56.00	197.30	8.26*10^−8^ CI (4.47*10^−8^–1.57*10^−7^)	
CH94-00	6	221.70	104.00	646.10	1.12*10^−8^ CI (5.20*10^−9^–3.27*10^−8^)	
CH00-10	10	416.90	171.10	3749.00	1.05*10^−8^ CI (4.31*10^−9^–9.45*10^−8^)	
CH78-10	32	597.70	342.40	1199.50	1.50*10^−7^ CI (8.59*10^−8^–3.01*10^−7^)	
VI85-87	2	68.00	23.60	588.30	NA	
VI87-89	2	75.00	27.30	526.30	3.10*10^−7^ CI (1.13*10^−7^–2.18*10^−6^)	
VI89-10	21	891.20	444.20	2496.40	1.47*10^-7^CI (7.35*10^−8^–4.13*10^−7^)	
VI85-10	25	666.70	247.50	2864.90	3.89*10^-7^CI (1.44*10^−7^–1.67*10^−6^)	

The TM3 coalescent likelihood-based method showed *N*_e_ between 161.15 and 2373.85 for the Chioggia time series (Table 2) and between 134.17 and 2883.85 for the Vieste time series (Table 2). For both sets of estimates, the overall *N*_e_ estimates were consistent with the magnitudes of shorter timeframes estimates.

**Table 2:** Temporal TM3 coalescent-based effective population size estimates. *N*_G_ = number of generations between a pair of samples. *N*_e_ = effective population size estimate. Lower and Upper 95% CI = represents a 95% Confidence Interval (CI) for *N*_e_ estimated for a pair of samples. Ne MAX = maximum prior effective population size defined in coalescent-based simulations. Values in bold represent the most likely coalescent estimates based on higher 95% CI limit per simulation.

		Ne MAX = 1000			Ne MAX = 5000			Ne MAX = 10000		
Timeframe	N_G_	*N*_e_	Lower 95% CI	Upper 95% CI	*N*_e_	Lower 95% CI	Upper 95% CI	*N*_e_	Lower 95% CI	Upper 95% CI
CH78-87	9	**282.79**	148.51	464.09	258.31	163.58	523.98	270.47	145.12	460.43
CH87-94	7	**161.15**	96.14	260.52	149.69	92.47	272.03	145.78	90.48	226.06
CH94-00	6	817.52	314.40	1000.00	1000.77	257.99	5000.00	**680.22**	0.00	10000.00
CH00-10	10	984.40	387.64	1000.00	1162.30	323.83	5000.00	**2373.85**	118.03	10000.00
CH78-10	32	950.69	529.09	1000.00	**1026.87**	493.44	4370.12	1086.84	419.73	4805.98
VI85-87	2	**182.23**	66.81	777.09	149.76	0.00	577.55	153.22	0.00	560.15
VI87-89	2	**134.17**	67.96	383.19	119.02	33.26	456.84	250.03	0.00	1591.39
VI89-10	21	991.57	752.78	1000.00	4675.32	1202.63	5000.00	**2883.85**	977.89	10000.00
VI85-10	25	990.87	823.19	1000.00	1740.50	812.00	5000.00	**1612.00**	890.08	5760.81

The results of the BOTTLENECK analysis using the IAM mutation model showed significant tests for heterozygosity excesses (*H*_exc_) in every sample for both “one-tail” and “two-tailed” Wilcoxon’s tests (Table 3). In contrast, tests using the SMM and TPM mutation models were not significance (Table 3). The Shift-Mode tests revealed normal L-shaped allele frequency distributions in all the samples.

**Table 3:** BOTTLENECK 1.2 results. IAM = Infinite Allele Model; TPM = Two Phase Mutation Model; SMM = Stepwise Mutation Model. *H*_def_ = One-tailed heterozygosity deficiency test; *H*_exc_ = One-tailed heterozygosity excess test; *H*_exc_*–H*_def_ = Two-tailed heterozygosity deficiency or excess test. Values in bold are significant P-values (*P* < 0.05).

Samples	IAM			TPM			SMM		
	H_def_	H_exc_	H_exc_-H_def_	H_def_	H_exc_	H_exc_-H_def_	H_def_	H_exc_	H_exc_-H_def_
**CH78**	0.9921	**0.0117**	**0.0234**	0.4687	0.5937	0.9375	0.2890	0.7656	0.5781
**CH 87**	0.9882	**0.0195**	**0.0390**	0.2343	0.8125	0.4687	0.1875	0.8515	0.3750
**CH94**	0.9960	**0.0078**	**0.0156**	0.7656	0.2890	0.5781	0.2890	0.7656	0.5781
**CH00**	0.9960	**0.0078**	**0.0156**	0.9609	0.0546	0.1093	0.8515	0.1875	0.3750
**CH10**	1.0000	**0.0039**	**0.0078**	0.5937	0.4687	0.9375	0.4687	0.5937	0.9375
**VI85**	1.0000	**0.0039**	**0.0078**	0.8515	0.1875	0.3750	0.6562	0.4062	0.8125
**VI87**	0.9960	**0.0078**	**0.0156**	0.4687	0.5937	0.9375	0.2890	0.7656	0.5781
**VI89**	1.0000	**0.0039**	**0.0078**	0.6562	0.4062	0.8125	0.4687	0.5937	0.9375
**VI10**	1.0000	**0.0039**	**0.0078**	0.6562	0.4062	0.8125	0.1875	0.8515	0.3750

**Fig 4:**
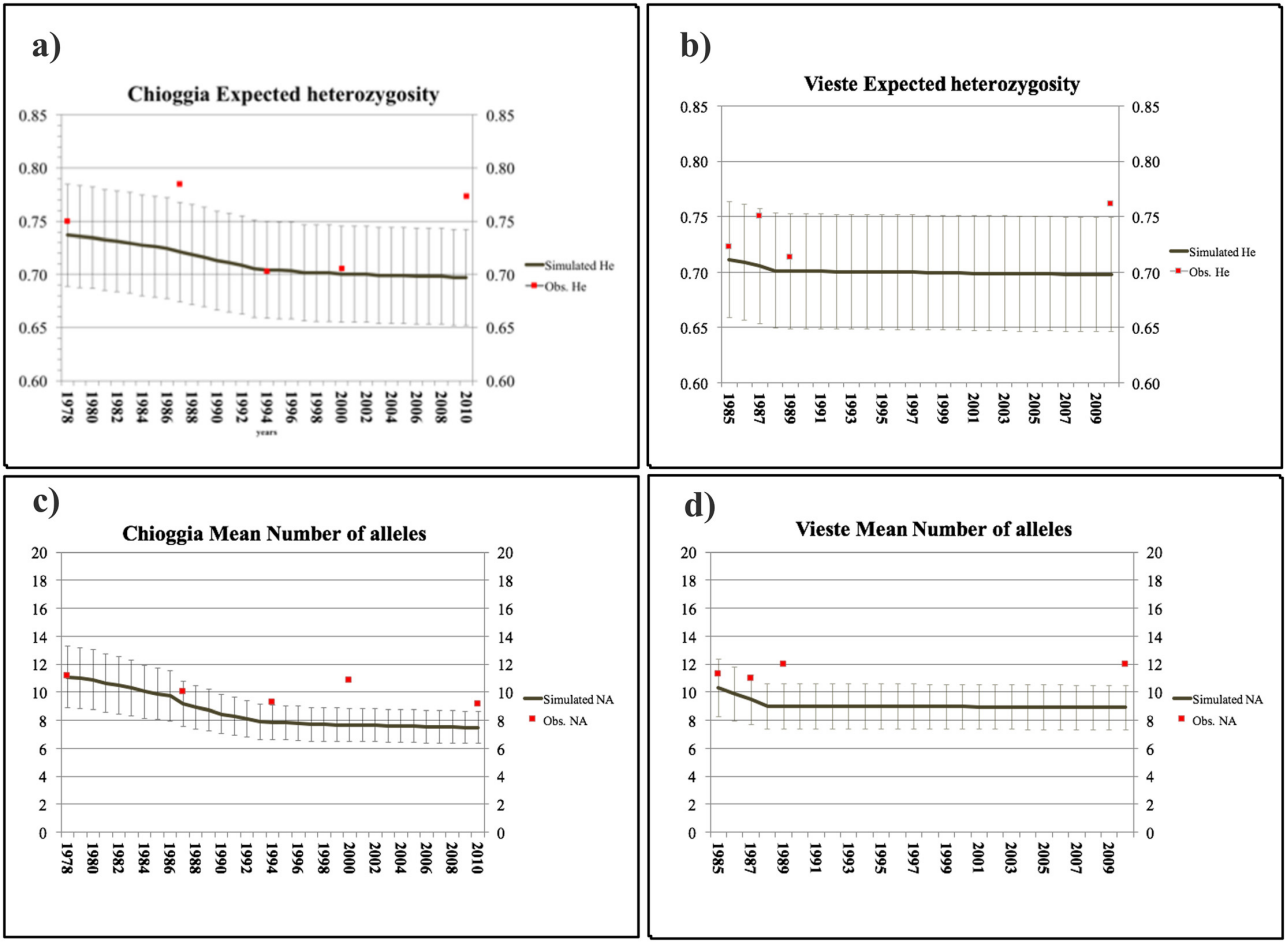
BOTTLESIM simulation. (**a**) Trend (solid line) of *H*_e_ and (**b**) *N*_A_ simulated values for a population (represented by CH78) experiencing a bottleneck for over 32 generations (years). Red squares represent the observed (**a**) *H*_e_ and (**b**) *N*_A_ values in CH78, CH87, CH94, CH00, and CH10. (**c**) Trend (solid line) of *H*_e_ and (**d**) *N*_A_ simulated values for a population (represented by VI85) that went through a bottleneck for over 25 generations (years). Red squares represent the observed (**c**) *H*_e_ and (**d**) *N*_A_ values in VI85, VI87, VI89, and VI10. Light grey bars are standard errors for simulated values.

Simulated population bottlenecks indicated the likely occurrence of bottlenecks in both the Chioggia and Vieste time series, based on comparisons between simulated and observed expected heterozygosity (*H*_e_) values. Similar values between simulated and observed *H*_e_ were found for CH78, CH94, and CH00 in the Chioggia time series (Fig 4a), whereas *H*_e_ values for CH87 and CH10 were outside the confidence interval (Fig 4a). In the Vieste time series, simulations showed similar simulated and observed *H*_e_ values for VI85, VI87, and VI89, whereas VI10 was placed outside the confidence interval (Fig 4b). Observed values of *N*_A_ were consistent with simulated *N*_A_ for CH78 and CH87 in the Chioggia time series and for VI85 and VI87 in the Vieste time series.

Estimates of θ_ST_ revealed 9 significant tests among 36 total pairwise comparisons (Table 4). These significant comparisons were largely between localities and years (i.e. significant tests were CH87-VI85, CH94-VI85, CH94-VI89, CH00-VI85, CH00-VI89, CH10-VI89) (Table 4). Genetic variation varied little with time within each time series. In fact, in the Chioggia time series only the CH94-CH00 comparison was statistically significant, whereas two tests (VI85-VI89 and VI85-VI10) were significant in the Vieste time series (Table 4).

**Table 4:** Pairwise multilocus estimates of θ_ST_. Bold values are significant after a sequential Bonferroni correction [42] for 36 multiple tests (*P* < 0.0014).

	**CH78**	**CH87**	**CH94**	**CH00**	**CH10**	**VI85**	**VI87**	**VI89**
**CH87**	0.0169							
**CH94**	0.0061	0.0198						
**CH00**	0.0154	0.016	**0.0048**					
**CH10**	0.0078	0.0069	0.0078	0.0074				
**VI85**	0.018	**0.0238**	**0.0107**	**0.0197**	0.0079			
**VI87**	0.0048	0.0217	0.0176	0.0233	0.0149	0.0186		
**VI89**	0.0139	0.022	**0.0078**	**0.0157**	**0.0189**	**0.0053**	0.0209	
**VI10**	0.0077	0.009	0.0049	0.0104	-0.0021	**0.0111**	0.0175	0.0052

STRUCTURE analysis showed a lack of genetic structure between and within the Chioggia and Vieste time series. The greatest likelihood indicated a single population, *K* = 1 [LnP(*K*) = -10843.28] (S1 Fig).

Outcomes from the neutrality tests are available in S1 File and in S5 and S6 Tables.

## Discussion

The extent of genetic variability estimated from both time series revealed the effectiveness of using archived DNA for population genetics studies. Otolith collections represent valuable sources of material for historical comparisons of genetic variation among locations or temporal variation within locations. Major challenges with the use of archived tissues are the reliability of results in light of potential DNA contamination and the level of polymorphism of the molecular marker. In the first case, we took considerable precautions to avoid DNA contamination. Genotypes were consistent between initial genotyping and re-genotyping some numerous individuals, indicating genotype quality. The amount of missing data was similar with that in other studies in which archived DNA from otoliths was used [6, 58–60]. In addition, the comparison between polymorphisms detected in the oldest and newest samples confirmed no age-related failure in genotyping. A new set of PCR primers for seven microsatellite loci yielding products < 200 bp produced a high degree of amplification success, with amplification success was between 68.75% and 100%. The number of loci genotyped in this study is similar to the mean number of loci typed in other archived DNA studies [6, 58–60].

### Loss of genetic variability as a consequence of demographic collapse

Temporal trends in genetic variables are concordant with abundance trends in the Chioggia and Vieste time series. However, the Chioggia time series showed greater changes in genetic variables than the Vieste time series. In the Chioggia time series, a significant reduction in *H*_e_ appeared after the population collapse in 1987 and lasted until 1994. This observation was additionally confirmed by a decrease in *N*_A_ from 1978 to 1994 and supports the idea of different responses of *H*_e_ and *N*_A_ to demographic declines [61]. After 2000, both *H*_e_ and *N*_A_ increased in the Chioggia time series in association with a growing population biomass in northern Adriatic Sea [22]. Values of *H*_e_ also declined in the middle-southern Adriatic basin (Vieste), but this drop was not statistically significant. However, values of *N*_A_ increased significantly after 1989. These results clearly show the influence of demographic fluctuations on genetic variability of Adriatic anchovies, which has also been observed in other marine populations [6, 62]. These results add to a growing number of studies showing a large contrast between the genetic effective population size and census in marine species [63].

Even though a significant reduction in *H*_e_ in northern Adriatic anchovies is consistent with a bottleneck in *N*_e_ and with a recent 10 to 20-fold drop in fishery biomass [19, 20, 22], the results from the heterozygosity excess test did not support this scenario. This outcome may be due to two mechanisms. First, the loss of genetic diversity after demographic decline may not have been strong enough to produce genetic profiles leading to heterozygosity excess. This weak-effect hypothesis, however, is inconsistent with the temporal drop in *H*_e_ in response to large declines in population abundance [64]. Second, the lack of significance may have been due to low statistical power of the test to detect heterozygosity excess with our dataset. Peery et al. [65] reviewed 703 populations belonging to 116 vertebrate species and concluded that even in species with strong population declines these tests often failed to detect bottlenecks [65]. The low statistical power of bottleneck tests results in many cases from little temporal variation in *N*_e_ [65], and this is consistent with our observations of a relatively small range of *N*_e_ among samples from Chioggia from 1978 to 2000, as well as that among samples from Vieste from 1985 to 1989.

The use of demographic simulations with BOTTLESIM showed that most of the observed *H*_e_ values between 1978 and 2000 in Chioggia and between 1985 and 1989 in Vieste, may be consistent with the loss of genetic variability as a consequence of a bottleneck. Observed *N*_A_ values between 1978 and 1987 in Chioggia and between 1985 and 1987 in Vieste were close to simulated values. These simulations also indicated a decrease in genetic diversity in the late 1980s that is consistent with bottlenecks in population size.

Reduced genetic variability as a consequence of a bottleneck, especially in the northern Adriatic Sea, is also indicated by significant deviations from Hardy-Weinberg expectations for Chioggia in 1978 and 1987, as a consequence of homozygote excess. These genetic signals support the hypothesis that a reduction in genetic variability began earlier than the decline in effective population size. Although unlikely in small pelagic fishes, other marine species characterized by high fecundities and a high variance in reproductive success (sweepstakes recruitment) often show signs of inbreeding [7, 66, 67].

### Evolutionary potential and effective population sizes in anchovies

The MCMC methods used to evaluate temporal variability in *N*_e_ in the Chioggia and the Vieste time series were characterized by good converges for this parameter, which gives some confidence in the results. We observed low levels of effective population sizes (*N*_e_) of only 100s of fish in the pre-collapse and early post-collapse periods in both historical time series. These results indicate that the reductions in genetic diversity were largely driven by genetic drift [13] and that genetic diversity dropped before the population collapse in 1987. Estimates of *N*_e_ from pre-collapse years were as low as those for 1987. Our global estimates of *N*_e_ for Adriatic anchovy indicate values of *N*_e_ between one hundred and a few thousand fish, values that have previously been reported in other small pelagics, such as European sardines [6, 12, 27], and in other marine organisms characterized by high fecundity, type III survivorship and sweepstakes recruitment [8, 62, 66]. These small effective population sizes challenge the idea that marine populations are invulnerable and inexhaustible resources by virtue of their huge census sizes [8, 12].

Estimates of *N*_e_/*N*_c_ in Adriatic anchovies was between 10^−6^ and 10^−8^ orders of magnitude and are smaller than those typical of many marine species (*N*_e_/*N*_c_ ≥ 10^−5^) [13]. These small ratios indicate that the large variance in reproductive success can lead to the demographic instability. Although the primary intent of this article was not to provide advice to management, it is nevertheless important to compare our temporal estimates of *N*_e_ with conservation guidelines provided by the revised 50/500 rule [68]. The 50/500 rule recommends that populations be maintained at sizes larger than 50 individuals over the short term and at least 500 individuals over the long term to avoid inbreeding and the loss of genetic diversity, and at sizes larger than 500–1000 to indefinitely retain evolutionary potential [reviewed in 68]. In our results, anchovy pre- and immediately post-population collapse showed estimates of *N*_e_ that come close to those recommended by 50/500 rules, suggesting that severe population declines place Adriatic anchovies at risk of losing evolutionary potential in the absence of stable connectivity among anchovy subpopulations in the Adriatic basin.

### Temporal population genetic integrity

Despite fluctuations in genetic variables in response to severe population declines, anchovies in the Adriatic Sea generally showed little genetic heterogeneity both between northern and middle-southern samples and among temporal samples in these areas. We detected a lack of genetic differentiation and a single population comprising all the Chioggia and Vieste samples. The occurrence a single genetic stock is also confirmed by a microsatellite study of samples from throughout the Adriatic basin (Ruggeri et al. in preparation). The lack of temporal variability confirms that this genetic composition has been maintained over at least the past four decades. Similar evidence was found in other temporal studies of small pelagics [6, 69] and other commercially exploited demersal fishes [58, 70]. The increasing trends in *H*_e_, *N*_A_ and *N*_e_ over time indicates that both localities have recovered from a population bottleneck, but that the recovery appears to have started earlier in Vieste (from 1989) than in Chioggia (from 1994). The relative geographic genetic homogeneity of anchovy populations in the Adriatic (Ruggeri et al. in preparation) may indicate that migration between subpopulations is important for the recovery of genetic diversity in local populations. In this way, migration between the Chioggia and Vieste populations likely led to the recovery of most of the genetic diversity lost in the Chioggia population over about seven generations.

## Conclusions

This study confirms the utility of archived DNA to address evolutionary and fishery-related problems. Our results indicate that, contrary to the common assumption that abundant species, such as anchovies, are not immune to the loss of genetic variability, because sweepstakes recruitment can lead to small effective population sizes. The effective population sizes of Adriatic anchovies are several orders of magnitude smaller than census population sizes. This large discrepancy indicates that anchovy populations are more vulnerable to fishery pressures and environmental change than previously thought, and these small effective sizes must be taken into account in the management of fishery harvests. Our results also indicate that temporal genetic data provides an assessment of the impact of demographic disturbances on the persistence of local populations.

## Supporting Information

S1 Fig Graphical plot of mean Ln[P(*K*)] from STRUCTURE [56, 57] simulation. (DOCX)

S1 File Describe the outliers`detection methods in details and provide results for these tests. (DOCX)

S1 Table Historical collection, specimens and data collection. (DOCX)

S2 Table Molecular markers information. Microsatellite loci primer sequences, reference genome and authors.
(DOCX)

S3 Table Locus-by-locus Probability of Identity (*P*_ID_) and Probability of Identity of Siblings (*P*_ID(sib)_) estimations [36]. (DOCX)

S4 Table Summary of genetic diversity observed at 7 microsatellite loci from the sampled archived materials. (DOCX)

S5 Table *F*_ST_ values and significances from the *fdist* [43] outlier detection test. (DOCX)

S6 Table Results from the LnRH method [45] to detect outlier loci. (DOCX)
