# Supplementary material for: Coupling Demographic and Genetic Variability from Archived Collections of European Anchovy (Engraulis encrasicolus)
Source: PLoS One. 2016 Mar 16;11(3):e0151507. doi: 10.1371/journal.pone.0151507 (PMC4794184; doi:10.1371/journal.pone.0151507)
Supplement: S1 Fig — (DOCX) [file pone.0151507.s001.docx]

S1 Fig


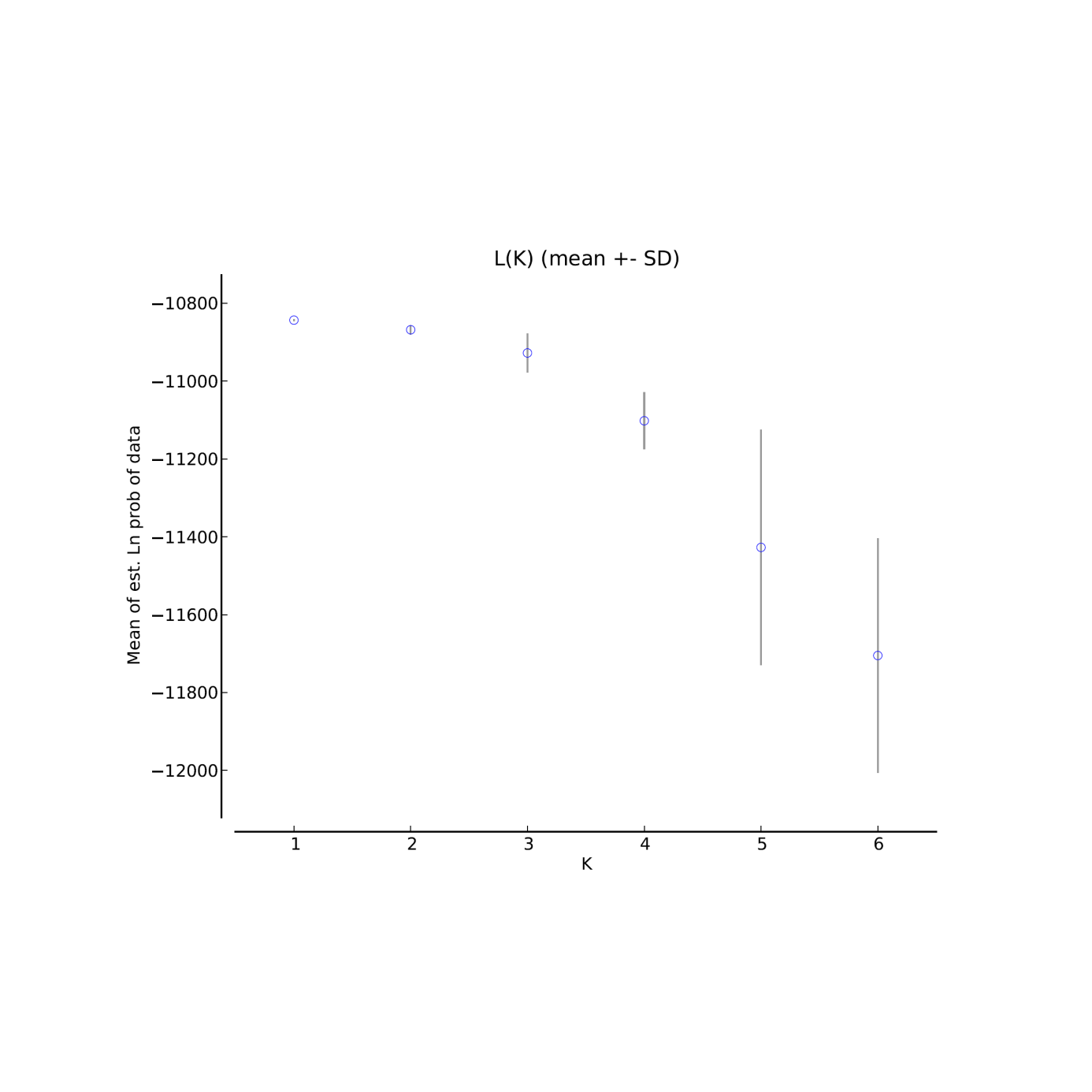


**S1 Fig.** Graphical plot of STRUCTURE [56-57] simulations results. Blue circles are mean Ln probability from each simulates K (1-6). Bars crossing blue circles explain the extent of standard deviations (SD) among replicates from each K simulated.
