# Supplementary material for: Coupling Demographic and Genetic Variability from Archived Collections of European Anchovy (Engraulis encrasicolus)
Source: PLoS One. 2016 Mar 16;11(3):e0151507. doi: 10.1371/journal.pone.0151507 (PMC4794184; doi:10.1371/journal.pone.0151507)
Supplement: S1 File — (DOCX) [file pone.0151507.s002.docx]

**Supporting Information to Ruggeri et al. (xxxx)**

**Methods to candidate outliers detection**

Detection of non-neutral distribution of allele frequencies was evaluated using two separate approaches: the *fdist* method [43] implemented in Lositan [44] and a microsatellite designed LnRH method [45]. The *fdist* method detects outliers loci using joint distributions of *F*_ST_ and expected heterozygosity *H*_e_, under an island migration model [43]. The *fdist* tests were conducted on the basis of 50,000 simulations under both IAM (Infinite Allele Model) and SMM (Stepwise Mutation Model) settings. Both tests were conducted on the whole dataset and subsequently, separating data, in two distinct temporal frames: before the demographic collapse (between 1978 and 1987) and after this event (between 1989 and 2010). These tests were conducted to define whether selection occurred before or after the demographic collapse in 1987 and to verify their neutrality.

The LnRH method compares genetic diversity estimates found in each locus between pairwise sampling locations applying the following formula:

LnRH_popA-popB_ = Ln [(1/(1−*H*e_popA_))^2^−1/(1/(1−*H*e_popB_))^2^−1]

The LnRH method assumes that microsatellite loci under selective constraints will display lowered levels of diversity [45]. Under neutral expectations LnRH values show a normal distribution [44], therefore, after data standardization (mean = 0, s. d. = 1), the 99% of neutral loci are expected to have LnRH values comprised within ±2.58. A Bonferroni correction [42] was applied to LnRH values (LnRH < -3.21 and LnRH > 3.21) with the aim to maintain a more conservative approach and avoid the identification of false positives.

**Candidate outliers detection results**

The *fdist* method under both IAM and SMM models revealed no outlier loci both when the whole dataset and the temporal frames before (between 1978 and 1987) and after the demographic collapse (between 1989 and 2010) were considered (S5 Table). The results from LnRH method showed a lack of significance in all the 36 LnRH pairwise comparisons between samples, suggesting even in this case no candidate outliers among loci used (Supplementary S6 Table).
