# Supplementary material for: Coupling Demographic and Genetic Variability from Archived Collections of European Anchovy (Engraulis encrasicolus)
Source: PLoS One. 2016 Mar 16;11(3):e0151507. doi: 10.1371/journal.pone.0151507 (PMC4794184; doi:10.1371/journal.pone.0151507)
Supplement: S1 Table — (DOCX) [file pone.0151507.s003.docx]

| **S1 Table** |  | | | | | |
| --- | --- | --- | --- | --- | --- | --- |
| **Historical collection** | | **Sample** | **year** | **Sampling months** | **N° of total analyzed individuals** | **N° of genotyped individuals** |
|  | | CH78 | 1978 | July-August | 59 | 48 |
|  | | CH87 | 1987 | May-June-July | 74 | 60 |
| CHIOGGIA | | CH94 | 1994 | May-June-July-September | 72 | 60 |
|  | | CH00 | 2000 | May-September | 68 | 60 |
|  | | CH10 | 2010 | July-August | 25 | 25 |
|  | | VI85 | 1985 | May-June | 69 | 60 |
| VIESTE | | VI87 | 1987 | May-June | 72 | 60 |
|  | | VI89 | 1989 | May-June-July-September | 70 | 60 |
|  | | VI10 | 2010 | July-August | 48 | 48 |

S1 Table. Table illustrating the historical collection to which belongs each sample analyzed, the sample identification code (Sample), the sampling year (year) and months (Sampling months) and the number of analyzed individuals per sample (N° of total analyzed individuals; the number includes individuals discarded from subsequent analysis due to contaminations or excess of missing data) and the number of genotyped individuals per sample (N° of genotyped individuals).
