# Supplementary material for: Coupling Demographic and Genetic Variability from Archived Collections of European Anchovy (Engraulis encrasicolus)
Source: PLoS One. 2016 Mar 16;11(3):e0151507. doi: 10.1371/journal.pone.0151507 (PMC4794184; doi:10.1371/journal.pone.0151507)
Supplement: S2 Table — Microsatellite loci primer sequences, reference genome and authors. (DOCX) [file pone.0151507.s004.docx]

| **S2 Table** | | | | | |
| --- | --- | --- | --- | --- | --- |
| **Locus name** | **Primers sequence (5'-3')** | **Repeated motif** | **Ta (°C)** | **Genome species** | **Authors** |
| Ee2-91b | F: GGCTTGAGCTTGGCATAGG | (CCGCA)_7_ | 60 | *E. encrasicolus* | [32] |
|  | R: CCGGAAGACACTCTGCACAC |  |  |  |  |
| Ee2-135 | F: AGGGCAGTGACAGGAGAGTC | (ATTAG)_10_ | 60 | *E. encrasicolus* | [32] |
|  | R: TCGTTACCCTGCGTTTATACTG |  |  |  |  |
| Ee2-508m | F:CCTGACAATGTTTCAAAGTTGG | (AGG)_8_ | 58 | *E. encrasicolus* | [32]* |
|  | R:CACCTGATGCTGCTTGGTAG |  |  |  |  |
| Ee2-407m | F:CCGGAGTTGGTAGCATCTGT | (CA)_13_ | 59 | *E. encrasicolus* | [32]* |
|  | R:GTGTGGGTCTGTGGGTGTTT |  |  |  |  |
| Ee2-165m | F:CTGGAACCTTCCTCGTTTTG | (CCT)_7_ | 58 | *E. encrasicolus* | [32]* |
|  | R:GGGATCTTCAGGGAACCAGT |  |  |  |  |
| Ee-10m | F:TCTGATTCTTGCCTTTGGCTA | [(GT)_9_CT]_2_[(GT)_2_CT]_3_ | 57 | *E. encrasicolus* | [31]* |
|  | R:ATGTTCTGGGGTGGCATAACT |  |  |  |  |
| Eja-183m | F:TTTGAATGGACACGATCATCA | (TCA)_13_ | 54 | *E. japonicus* | [33]* |
|  | R:TAAGGCCCCCTATCCAATGT |  |  |  |  |
|  |  |  |  |  |  |

S2 Table. Information concerning the molecular markers employed in this study. Ta (°C) refers to annealing temperature expressed in Celsius degrees. * denotes newly designed primer sequences if compared to the original primer sequences described in the reference papers.
