# Supplementary material for: Coupling Demographic and Genetic Variability from Archived Collections of European Anchovy (Engraulis encrasicolus)
Source: PLoS One. 2016 Mar 16;11(3):e0151507. doi: 10.1371/journal.pone.0151507 (PMC4794184; doi:10.1371/journal.pone.0151507)
Supplement: S3 Table — (DOCX) [file pone.0151507.s005.docx]

| **S3 Table** |  |  |  |  |  |  |  |  |  |
| --- | --- | --- | --- | --- | --- | --- | --- | --- | --- |
| ***P*_ID_ by Locus** |  |  |  |  |  |  |  |  |  |
| **Pop** | **N** | **Ee91b** | **Ee165m** | **Ee135** | **Ee508m** | **Ee407m** | **Ee10m** | **Eja183** | *P*_ID_ |
| CH78 | 48 | 0.091 | 0.252 | 0.037 | 0.222 | 0.025 | 0.126 | 0.046 | 2.820E-08 |
| CH87 | 60 | 0.085 | 0.190 | 0.042 | 0.146 | 0.026 | 0.053 | 0.057 | 7.731E-09 |
| CH94 | 60 | 0.105 | 0.273 | 0.047 | 0.274 | 0.097 | 0.146 | 0.055 | 2.854E-07 |
| CH00 | 60 | 0.117 | 0.235 | 0.037 | 0.223 | 0.155 | 0.123 | 0.062 | 2.662E-07 |
| CH10 | 25 | 0.084 | 0.154 | 0.032 | 0.168 | 0.104 | 0.035 | 0.085 | 2.130E-08 |
| VI85 | 60 | 0.078 | 0.238 | 0.041 | 0.267 | 0.043 | 0.161 | 0.042 | 6.065E-08 |
| VI87 | 60 | 0.085 | 0.203 | 0.036 | 0.276 | 0.010 | 0.142 | 0.052 | 1.329E-08 |
| VI89 | 60 | 0.061 | 0.348 | 0.044 | 0.276 | 0.034 | 0.165 | 0.034 | 4.860E-08 |
| VI10 | 48 | 0.073 | 0.253 | 0.036 | 0.186 | 0.043 | 0.053 | 0.054 | 1.535E-08 |
|  |  |  |  |  |  |  |  |  |  |
| ***P*_ID(sib)_ by Locus** |  |  |  |  |  |  |  |  |  |
| **Pop** | **N** | **Ee91b** | **Ee165m** | **Ee135** | **Ee508m** | **Ee407m** | **Ee10m** | **Eja183** | *P*_ID(sib)_ |
| CH78 | 48 | 0.391 | 0.519 | 0.331 | 0.526 | 0.322 | 0.431 | 0.344 | 1.682E-03 |
| CH87 | 60 | 0.384 | 0.490 | 0.337 | 0.437 | 0.324 | 0.356 | 0.355 | 1.133E-03 |
| CH94 | 60 | 0.402 | 0.542 | 0.344 | 0.558 | 0.412 | 0.449 | 0.354 | 2.743E-03 |
| CH00 | 60 | 0.417 | 0.513 | 0.330 | 0.507 | 0.475 | 0.435 | 0.362 | 2.674E-03 |
| CH10 | 25 | 0.382 | 0.448 | 0.324 | 0.475 | 0.417 | 0.337 | 0.384 | 1.419E-03 |
| VI85 | 60 | 0.378 | 0.527 | 0.337 | 0.559 | 0.353 | 0.467 | 0.339 | 2.095E-03 |
| VI87 | 60 | 0.385 | 0.484 | 0.331 | 0.558 | 0.291 | 0.459 | 0.351 | 1.609E-03 |
| VI89 | 60 | 0.361 | 0.611 | 0.340 | 0.560 | 0.339 | 0.475 | 0.328 | 2.222E-03 |
| VI10 | 48 | 0.372 | 0.543 | 0.329 | 0.488 | 0.344 | 0.361 | 0.354 | 1.425E-03 |

S3 Table. Table reporting Probability of Identity (*P*_ID_) and Probability of Identity of Siblings (*P*_ID(sib)_) estimations [36]. The estimates are produced both independently for every single locus and as complete set of loci analyzed from each sampling site/year.
