# Supplementary material for: Coupling Demographic and Genetic Variability from Archived Collections of European Anchovy (Engraulis encrasicolus)
Source: PLoS One. 2016 Mar 16;11(3):e0151507. doi: 10.1371/journal.pone.0151507 (PMC4794184; doi:10.1371/journal.pone.0151507)
Supplement: S4 Table — (DOCX) [file pone.0151507.s006.docx]

| **S4 Table** | | | | | | | | | | | | | |
| --- | --- | --- | --- | --- | --- | --- | --- | --- | --- | --- | --- | --- | --- |
|  |  |  | **CH78** | **CH87** | **CH94** | **CH00** | **CH10** | **VI85** | **VI87** | **VI89** | **VI10** |  | **TOT** |
| **Ee2-91b** | ***N_A_*** |  | 7 | 7 | 6 | 8 | 6 | 8 | 8 | 9 | 9 |  | 11 |
|  | ***N*** |  | 33 | 51 | 57 | 55 | 24 | 58 | 56 | 59 | 48 |  |  |
|  | ***f_null_*** |  | 0.067 | 0.046 | -0.034 | -0.033 | 0.063 | -0.006 | 0.043 | -0.003 | 0.035 |  |  |
|  | ***H*_e_** |  | 0.778 | 0.784 | 0.755 | 0.732 | 0.800 | 0.790 | 0.780 | 0.815 | 0.800 |  |  |
|  | ***H*_o_** |  | 0.643 | 0.692 | 0.807 | 0.782 | 0.667 | 0.793 | 0.696 | 0.814 | 0.729 |  |  |
|  | ***F_IS_*** |  | 0.176 | 0.118 | -0.070 | -0.069 | 0.171 | -0.004 | 0.109 | 0.001 | 0.090 |  |  |
|  | ***R_S_*** |  | 6.699 | 6.288 | 5.364 | 6.311 | 6.000 | 6.348 | 6.991 | 7.619 | 7.067 |  | 6.728 |
|  |  |  |  |  |  |  |  |  |  |  |  |  |  |
| **Ee2-165m** | ***N_A_*** |  | 5 | 6 | 4 | 4 | 4 | 4 | 4 | 5 | 5 |  | 7 |
|  | ***N*** |  | 48 | 58 | 60 | 59 | 25 | 59 | 60 | 60 | 46 |  |  |
|  | ***f_null_*** |  | -0.049 | -0.079 | -0.180 | -0.118 | 0.072 | -0.062 | -0.081 | -0.068 | 0.040 |  |  |
|  | ***H*_e_** |  | 0.595 | 0.620 | 0.558 | 0.597 | 0.695 | 0.569 | 0.639 | 0.455 | 0.546 |  |  |
|  | ***H*_o_** |  | 0.667 | 0.741 | 0.833 | 0.780 | 0.560 | 0.661 | 0.767 | 0.550 | 0.478 |  |  |
|  | ***F_IS_*** |  | -0.123 | -0.198 | **-0.500** | **-0.308** | 0.197 | -0.162 | -0.202 | -0.210 | 0.125 |  |  |
|  | ***R_S_*** |  | 4.081 | 4.813 | 3.626 | 3.872 | 4.000 | 3.953 | 3.949 | 3.849 | 4.309 |  | 4.098 |
|  |  |  |  |  |  |  |  |  |  |  |  |  |  |
| **Ee2-135** | ***N_A_*** |  | 9 | 9 | 13 | 11 | 10 | 11 | 11 | 11 | 11 |  | 14 |
|  | ***N*** |  | 46 | 52 | 56 | 58 | 25 | 56 | 51 | 58 | 47 |  |  |
|  | ***f_null_*** |  | 0.058 | 0.042 | 0.007 | -0.012 | 0.090 | 0.033 | 0.007 | 0.017 | 0.040 |  |  |
|  | ***H*_e_** |  | 0.869 | 0.856 | 0.842 | 0.865 | 0.890 | 0.854 | 0.865 | 0.848 | 0.870 |  |  |
|  | ***H*_o_** |  | 0.750 | 0.769 | 0.821 | 0.879 | 0.700 | 0.786 | 0.843 | 0.810 | 0.786 |  |  |
|  | ***F_IS_*** |  | 0.139 | 0.102 | 0.025 | -0.016 | 0.218 | 0.081 | 0.026 | 0.045 | 0.098 |  |  |
|  | ***R_S_*** |  | 8.229 | 8.177 | 9.515 | 8.682 | 9.791 | 9.064 | 9.163 | 9.138 | 8.851 |  | 9.273 |
|  |  |  |  |  |  |  |  |  |  |  |  |  |  |
| **Ee2-508m** | ***N_A_*** |  | 8 | 6 | 7 | 9 | 7 | 8 | 7 | 6 | 9 |  | 12 |
|  | ***N*** |  | 41 | 57 | 59 | 60 | 25 | 58 | 57 | 60 | 48 |  |  |
|  | ***f_null_*** |  | 0.068 | 0.076 | -0.014 | 0.020 | -0.003 | -0.013 | -0.003 | -0.011 | **0.135** |  |  |
|  | ***H*_e_** |  | 0.569 | 0.709 | 0.526 | 0.603 | 0.647 | 0.519 | 0.527 | 0.522 | 0.628 |  |  |
|  | ***H*_o_** |  | 0.455 | 0.568 | 0.542 | 0.567 | 0.640 | 0.534 | 0.526 | 0.533 | 0.400 |  |  |
|  | ***F_IS_*** |  | 0.203 | 0.201 | -0.031 | 0.061 | 0.012 | -0.030 | 0.001 | -0.023 | 0.367 |  |  |
|  | ***R_S_*** |  | 6.338 | 5.096 | 5.108 | 5.577 | 6.497 | 5.431 | 4.463 | 4.781 | 7.273 |  | 5.562 |
|  |  |  |  |  |  |  |  |  |  |  |  |  |  |
| **Ee2-407m** | ***N_A_*** |  | 22 | 21 | 11 | 14 | 11 | 24 | 21 | 25 | 17 |  | 38 |
|  | ***N*** |  | 33 | 48 | 36 | 49 | 25 | 47 | 23 | 58 | 48 |  |  |
|  | ***f_null_*** |  | **0.059** | **0.125** | 0.065 | 0.047 | -0.048 | **0.078** | **0.126** | 0.053 | 0.012 |  |  |
|  | ***H*_e_** |  | 0.883 | 0.877 | 0.734 | 0.634 | 0.733 | 0.826 | 0.945 | 0.847 | 0.843 |  |  |
|  | ***H*_o_** |  | 0.759 | 0.632 | 0.611 | 0.551 | 0.800 | 0.674 | 0.682 | 0.741 | 0.813 |  |  |
|  | ***F_IS_*** |  | 0.143 | **0.282** | 0.169 | 0.132 | -0.093 | 0.185 | **0.283** | 0.125 | 0.037 |  |  |
|  | ***R_S_*** |  | 16.722 | 15.105 | 8.979 | 9.025 | 9.432 | 14.676 | 18.995 | 15.063 | 11.161 |  | 13.784 |
|  |  |  |  |  |  |  |  |  |  |  |  |  |  |
|  |  |  | **CH78** | **CH87** | **CH94** | **CH00** | **CH10** | **VI85** | **VI87** | **VI89** | **VI10** |  | **TOT** |
| **Ee2-10m** | ***N_A_*** |  | 12 | 11 | 13 | 17 | 18 | 13 | 13 | 12 | 17 |  | 26 |
|  | ***N*** |  | 46 | 57 | 60 | 60 | 25 | 60 | 59 | 60 | 48 |  |  |
|  | ***f_null_*** |  | 0.055 | **0.074** | -0.055 | -0.065 | 0.024 | 0.039 | 0.026 | -0.020 | -0.004 |  |  |
|  | ***H*_e_** |  | 0.709 | 0.824 | 0.680 | 0.696 | 0.861 | 0.652 | 0.659 | 0.639 | 0.813 |  |  |
|  | ***H*_o_** |  | 0.609 | 0.681 | 0.767 | 0.800 | 0.800 | 0.583 | 0.610 | 0.667 | 0.813 |  |  |
|  | ***F_IS_*** |  | 0.143 | 0.176 | -0.129 | -0.150 | 0.072 | 0.106 | 0.075 | -0.044 | 0.001 |  |  |
|  | ***R_S_*** |  | 8.043 | 8.999 | 7.679 | 8.945 | 14.939 | 7.131 | 8.388 | 7.096 | 11.818 |  | 9.786 |
|  |  |  |  |  |  |  |  |  |  |  |  |  |  |
| **Eja183m** | ***N_A_*** |  | 15 | 10 | 11 | 13 | 8 | 11 | 13 | 16 | 16 |  | 21 |
|  | ***N*** |  | 39 | 53 | 59 | 58 | 25 | 59 | 49 | 58 | 46 |  |  |
|  | ***f_null_*** |  | 0.008 | 0.041 | 0.004 | -0.030 | -0.037 | 0.035 | 0.005 | -0.001 | 0.056 |  |  |
|  | ***H*_e_** |  | 0.847 | 0.827 | 0.827 | 0.815 | 0.791 | 0.851 | 0.834 | 0.868 | 0.828 |  |  |
|  | ***H*_o_** |  | 0.821 | 0.745 | 0.814 | 0.862 | 0.840 | 0.780 | 0.816 | 0.862 | 0.717 |  |  |
|  | ***F_IS_*** |  | 0.031 | 0.101 | 0.017 | -0.058 | -0.063 | 0.085 | 0.021 | 0.007 | 0.135 |  |  |
|  | ***R_S_*** |  | 10.213 | 8.183 | 8.603 | 9.283 | 7.329 | 9.381 | 9.432 | 11.019 | 10.437 |  | 9.808 |
|  |  |  |  |  |  |  |  |  |  |  |  |  |  |
| ***Average*** | ***N_AM_*** |  | 11.143 | 10.000 | 9.286 | 10.857 | 9.143 | 11.286 | 11.000 | 12.000 | 12.000 |  |  |
|  | ***H*_e_** |  | 0.737 | 0.767 | 0.703 | 0.706 | 0.767 | 0.717 | 0.746 | 0.713 | 0.753 |  |  |
|  | ***H*_o_** |  | 0.622 | 0.590 | 0.742 | 0.746 | 0.671 | 0.679 | 0.684 | 0.711 | 0.643 |  |  |
|  | ***F_IS_*** |  | **0.106** | **0.123** | -0.056 | -0.057 | 0.078 | 0.050 | 0.060 | 0.003 | **0.113** |  |  |
|  | ***R_S_*** |  | 8.618 | 8.094 | 6.982 | 7.385 | 8.284 | 7.998 | 8.769 | 8.366 | 8.702 |  | 8.434 |
|  |  |  |  |  |  |  |  |  |  |  |  |  |  |

S4 Table. Summary of genetic variability observed at 7 microsatellite loci from the sampled archived materials. *N*_A_, number of alleles observed per location; *N*, number of individuals correctly genotyped; *N*_AM_, mean number of alleles observed per location; *f_null_,* null allele frequency calculated with [39] formula; *H*_o_, observed heterozygosity; *H*_e_, expected heterozygosity; *F*_IS_, inbreeding coefficient estimates. *R*_S_, allelic richness estimates standardized at 41 individuals. Bold *F*_IS_ values are significant (<0.05) after a sequential Bonferroni correction [42]. Bold *f_null_* values are those exceed the 5% null allele tolerance threshold. Asterisk (*) denotes estimates still showed null allele signals after [39] method.
