# Supplementary material for: Coupling Demographic and Genetic Variability from Archived Collections of European Anchovy (Engraulis encrasicolus)
Source: PLoS One. 2016 Mar 16;11(3):e0151507. doi: 10.1371/journal.pone.0151507 (PMC4794184; doi:10.1371/journal.pone.0151507)
Supplement: S5 Table — (DOCX) [file pone.0151507.s007.docx]

|  | | | | | | | |
| --- | --- | --- | --- | --- | --- | --- | --- |
| **S5 Table** |  |  |  |  |  |  |  |
|  |  |  |  | **IAM** |  |  |  |
| **Locus** | ***F*_ST-TOT_** | ***F*_ST-CH-TOT_** | ***F*_ST-VI-TOT_** | ***F*_ST-CH78-87_** | ***F*_ST-CH94-10_** | ***F*_ST-VI85-87_** | ***F*_ST-VI89-10_** |
|  |  |  |  |  |  |  |  |
| **Ee2-91b** | 0.0055 | 0.0034 | 0.0059 | 0.0170 | -0.0003 | 0.0104 | 0.0004 |
| **Ee2-165m** | 0.0273 | 0.0083 | 0.0618 | 0.0312 | 0.0023 | 0.0837 | 0.0023 |
| **Ee2-135** | 0.0029 | 0.0019 | 0.0033 | -0.0013 | 0.0053 | 0.0011 | 0.0109 |
| **Ee2-508m** | 0.0162 | 0.0259 | -0.0017 | 0.0733 | 0.0056 | -0.0035 | -0.0011 |
| **Ee2-407m** | 0.0197 | 0.0233 | 0.0093 | -0.0016 | 0.0044 | 0.0232 | 0.0006 |
| **Ee-10m** | 0.0264 | 0.0218 | 0.0274 | 0.0189 | 0.0292 | 0.0332 | 0.0223 |
| **Eja183m** | 0.0004 | -0.0021 | 0.0008 | 0.0071 | -0.0064 | -0.0064 | 0.0015 |
|  |  |  |  |  |  |  |  |
|  |  |  |  | **SMM** |  |  |  |
| **Locus** | ***F*_ST-TOT_** | ***F*_ST-CH-TOT_** | ***F*_ST-VI-TOT_** | ***F*_ST-CH78-87_** | ***F*_ST-CH94-10_** | ***F*_ST-VI85-87_** | ***F*_ST-VI89-10_** |
|  |  |  |  |  |  |  |  |
| **Ee2-91b** | 0.0055 | 0.0034 | 0.0059 | 0.017 | -0.0003 | 0.0104 | 0.0004 |
| **Ee2-165m** | 0.0273 | 0.0083 | 0.0618 | 0.0312 | 0.003 | 0.0837 | 0.0023 |
| **Ee2-135** | 0.0029 | 0.0019 | 0.0033 | -0.0013 | 0.0053 | 0.0011 | 0.0109 |
| **Ee2-508m** | 0.0162 | 0.0259 | -0.0017 | 0.0733 | 0.0056 | -0.0035 | -0.0011 |
| **Ee2-407m** | 0.0197 | 0.0233 | 0.0093 | -0.0016 | 0.0044 | 0.0233 | 0.0006 |
| **Ee-10m** | 0.0264 | 0.0218 | 0.0274 | 0.0189 | 0.0293 | 0.0332 | 0.0223 |
| **Eja183m** | 0.0004 | -0.0021 | 0.0008 | 0.0071 | -0.0064 | -0.0064 | 0.0015 |
|  |  |  |  |  |  |  |  |

S5 Table. Results from the *fdist* [43] outlier loci detection tests. IAM = Infinite Allele Mutation model; SMM = Stepwise Mutation Model. *F*_ST-TOT_ = mean *F*_ST_ value among all samples analyzed; *F*_ST-CH-TOT_ = mean *F*_ST_ value among all Chioggia samples analyzed; *F*_ST-VI-TOT_ = mean *F*_ST_ value among all Vieste samples analyzed; *F*_ST-CH78-87_ = mean *F*_ST_ value among all Chioggia samples before 1987; *F*_ST-CH94-10_ = mean *F*_ST_ value among all Chioggia samples after 1987; *F*_ST-VI85-87_ = mean *F*_ST_ value among all Vieste samples before 1987; *F*_ST-VI89-10_ = mean *F*_ST_ value among all Vieste samples after 1987. Significant values for selection were denoted by: * < 0.05; ** < 0.01; *** < 0.001.
