# Supplementary material for: Coupling Demographic and Genetic Variability from Archived Collections of European Anchovy (Engraulis encrasicolus)
Source: PLoS One. 2016 Mar 16;11(3):e0151507. doi: 10.1371/journal.pone.0151507 (PMC4794184; doi:10.1371/journal.pone.0151507)
Supplement: S6 Table — (DOCX) [file pone.0151507.s008.docx]

| **S6 Table** |  |  |  |  |  |  |  |  |
| --- | --- | --- | --- | --- | --- | --- | --- | --- |
| Locus | **CH78-CH87** | **CH78-CH94** | **CH78-CH00** | **CH78-CH10** | **CH78-VI85** | **CH78-VI87** | **CH78-VI89** | **CH78-VI10** |
| **Ee2-91b** | -0.0027 | 0.0114 | 0.0242 | -0.0098 | -0.0054 | -0.0010 | -0.0156 | -0.0098 |
| **Ee2-165b** | -0.0236 | 0.0379 | -0.0028 | -0.0818 | 0.0254 | -0.0401 | 0.1721 | 0.0516 |
| **Ee2-135m** | 0.0037 | 0.0079 | 0.0010 | -0.0051 | 0.0041 | 0.0010 | 0.0059 | -0.0003 |
| **Ee2-508m** | -0.1170 | 0.0485 | -0.0343 | -0.0729 | 0.0575 | 0.0476 | 0.0542 | -0.0570 |
| **Ee2-407m** | 0.0016 | 0.0599 | 0.1302 | 0.0600 | 0.0171 | -0.0107 | 0.0101 | 0.0112 |
| **Ee2-10m** | -0.0570 | 0.0200 | 0.0084 | -0.0689 | 0.0407 | 0.0352 | 0.0516 | -0.0528 |
| **Eja-183m** | 0.0065 | 0.0065 | 0.0111 | 0.0209 | -0.0014 | 0.0043 | -0.0062 | 0.0061 |
| Locus | **CH87-CH94** | **CH87-CH00** | **CH87-CH10** | **CH87-VI85** | **CH87-VI87** | **CH87-VI89** | **CH87-VI10** | **CH94-CH00** |
| **Ee2-91b** | 0.0141 | 0.0269 | -0.0070 | -0.0026 | 0.0018 | -0.0129 | -0.0071 | 0.0128 |
| **Ee2-165b** | 0.0615 | 0.0208 | -0.0582 | 0.0490 | -0.0164 | 0.1957 | 0.0752 | -0.0407 |
| **Ee2-135m** | 0.0042 | -0.0027 | -0.0088 | 0.0004 | -0.0027 | 0.0022 | -0.0040 | -0.0069 |
| **Ee2-508m** | 0.1656 | 0.0827 | 0.0441 | 0.1746 | 0.1646 | 0.1712 | 0.0600 | -0.0829 |
| **Ee2-407m** | 0.0583 | 0.1286 | 0.0586 | 0.0156 | -0.0123 | 0.0085 | 0.0096 | 0.0703 |
| **Ee2-10m** | 0.0770 | 0.0654 | -0.0119 | 0.0977 | 0.0922 | 0.1086 | 0.0042 | -0.0116 |
| **Eja-183m** | -0.0001 | 0.0046 | 0.0144 | -0.0080 | -0.0022 | -0.0128 | -0.0004 | 0.0047 |
| Locus | **CH94-CH10** | **CH94-VI85** | **CH94-VI87** | **CH94-VI89** | **CH94-VI10** | **CH00-CH10** | **CH00-VI85** | **CH00-VI87** |
| **Ee2-91b** | -0.0212 | -0.0168 | -0.0124 | -0.0270 | -0.0212 | -0.0339 | -0.0296 | -0.0251 |
| **Ee2-165b** | -0.1197 | -0.0125 | -0.0780 | 0.1342 | 0.0137 | -0.0790 | 0.0282 | -0.0373 |
| **Ee2-135m** | -0.0130 | -0.0038 | -0.0069 | -0.0020 | -0.0082 | -0.0061 | 0.0031 | 0.0000 |
| **Ee2-508m** | -0.1215 | 0.0090 | -0.0010 | 0.0056 | -0.1056 | -0.0386 | 0.0918 | 0.0819 |
| **Ee2-407m** | 0.0003 | -0.0428 | -0.0706 | -0.0498 | -0.0487 | -0.0700 | -0.1130 | -0.1409 |
| **Ee2-10m** | -0.0889 | 0.0207 | 0.0152 | 0.0316 | -0.0728 | -0.0773 | 0.0323 | 0.0268 |
| **Eja-183m** | 0.0144 | -0.0079 | -0.0021 | -0.0127 | -0.0004 | 0.0098 | -0.0125 | -0.0068 |
| Locus | **CH00-VI89** | **CH00-VI10** | **CH10-VI85** | **CH10-VI87** | **CH10-VI89** | **CH10-VI10** | **VI85-VI87** | **VI85-VI89** |
| **Ee2-91b** | -0.0398 | -0.0340 | 0.0044 | 0.0088 | -0.0059 | -0.0001 | 0.0044 | -0.0103 |
| **Ee2-165b** | 0.1749 | 0.0544 | 0.1072 | 0.0417 | 0.2539 | 0.1334 | -0.0654 | 0.1467 |
| **Ee2-135m** | 0.0049 | -0.0013 | 0.0092 | 0.0061 | 0.0110 | 0.0048 | -0.0031 | 0.0018 |
| **Ee2-508m** | 0.0885 | -0.0227 | 0.1305 | 0.1205 | 0.1271 | 0.0159 | -0.0009 | -0.0033 |
| **Ee2-407m** | -0.1201 | -0.1190 | -0.0431 | -0.0709 | -0.0501 | -0.0490 | -0.0278 | -0.0070 |
| **Ee2-10m** | 0.0432 | -0.0612 | 0.1096 | 0.1041 | 0.1205 | 0.0161 | -0.0055 | 0.0109 |
| **Eja-183m** | -0.0174 | -0.0050 | -0.0223 | -0.0166 | -0.0271 | -0.0148 | 0.0057 | -0.0048 |
| Locus | **VI85-VI10** | **VI87-VI89** | **VI87-VI10** | **VI89-VI10** |  |  |  |  |
| **Ee2-91b** | -0.0045 | -0.0147 | -0.0089 | 0.0058 |  |  |  |  |
| **Ee2-165b** | 0.0262 | 0.2121 | 0.0917 | -0.1205 |  |  |  |  |
| **Ee2-135m** | -0.0044 | 0.0049 | -0.0013 | -0.0062 |  |  |  |  |
| **Ee2-508m** | -0.1145 | 0.0066 | -0.1046 | -0.1112 |  |  |  |  |
| **Ee2-407m** | -0.0060 | 0.0208 | 0.0219 | 0.0011 |  |  |  |  |
| **Ee2-10m** | -0.0935 | 0.0164 | -0.0880 | -0.1044 |  |  |  |  |
| **Eja-183m** | 0.0075 | -0.0106 | 0.0018 | 0.0123 |  |  |  |  |
|  |  |  |  |  |  |  |  |  |

**S6 Table.** Results from the LnRH method [45] to detect outlier loci. Significant values (LnRH < -3.21 and LnRH > 3.21) were highlighted in bold.
